# Postpolypectomy Bleeding Prevention and More Complete Precancerous Colon Polyp Removal With Endoscopic Mucosal Stripping (EMS)

**DOI:** 10.3389/fmed.2018.00312

**Published:** 2018-11-08

**Authors:** Zongyu John Chen, Kenneth P. Batts

**Affiliations:** ^1^Minnesota Gastroenterology, Minneapolis, MN, United States; ^2^Hospital Pathology Associates, Minneapolis, MN, United States

**Keywords:** endoscopic mucosal stripping (EMS), polypectomy, post-polypectomy bleeding, colonoscopy quality and safety, colon cancer prevention

## Abstract

**Background and Aims:** Postpolypectomy bleeding and incomplete polyp removal are important complication and quality concerns of colonoscopy for colon cancer prevention. We investigated if endoscopic mucosal stripping (EMS) as a technical modification of traditional cold snare polypectomy to avoid submucosal injury during removal of non-pedunculated colon polyps could prevent postpolypectomy bleeding and facilitate complete polyp removal.

**Methods:** This is an Internal Review Board exemption-granted retrospective analysis of 5,142 colonoscopies with snare polypectomy performed by one of the authors (ZJC) at Minnesota Gastroenterology ambulatory endoscopy centers during a 12-year period divided into pre-EMS era (2005–2012, *n* = 2,973) and EMS era (2013–2016, *n* = 2169) with systemic adoption of EMS starting 2013. Change in postpolypectomy bleeding rate before and after EMS adoption and EMS polypectomy completeness were evaluated.

**Results:** Zero postpolypectomy bleeding case was found during EMS era (rate 0%) compared with 10 bleeding cases during pre-EMS era (rate 0.336%). This difference was statistically significant (*P* = 0.0055) and remained so after excluding 2 bleeding cases of pedunculated polyps (*P* = 0.012). All bleeding cases involved hot snare polypectomy. Histological examination of the involved polyps showed substantial submucosal vascular damage in contrast to a remarkable paucity of submucosa in comparable advanced polyps removed using EMS. Both biopsy and follow-up colonoscopy examination of the polypectomy sites confirmed that EMS more completely removed non-pedunculated advanced polyps.

**Conclusions:** EMS polypectomy was effective in preventing postpolypectomy bleeding and facilitated complete polyp removal.

Colonoscopy for colon cancer screening is effective at both mortality reduction through early detection and prevention through removal of precancerous polyps (1–8). These benefits, however, could be offset by colonoscopy quality inadequacy and complications (9–17). Missed polyps and incomplete polypectomy may contribute to interval cancer development (cancers detected after colonoscopy) (10–14, 18, 19). Although post-colonoscopy hemorrhage is relatively uncommon, occurring in 1–6/1000, (15–17) it is the leading complication resulting in unplanned hospital visits within 7 days of colonoscopy (20). Polypectomy is the overwhelming contributor (21–26). Risk factors that increase postpolypectomy bleeding include polyp size, number of polyps, anti-coagulation therapy and polyp histology (17, 21, 27, 28). Advanced polypectomy techniques such as endoscopic mucosal resection (EMR) and endoscopic submucosal dissection (ESD) appear to more completely remove polyps but also increase procedure time, cost and bleeding risks (17, 29–31).

Aside from EMR and ESD, little attention has been given to polypectomy technique as a risk factor for postpolypectomy bleeding. Indeed, the generally accepted and widely used traditional snare polypectomy technique to guillotine the ensnared polyp with or without heat coagulation has not changed much for nearly 5 decades (32–37). To prevent postpolypectomy bleeding, one of the authors (ZJC) developed endoscopic mucosal stripping (EMS) as a modification of traditional cold snare polypectomy (TCSP) to avoid cutting into blood vessel-rich submucosa because all non-cancerous polyps which are the most frequent type of polyps seen during screening colonoscopy are by definition confined to mucosa and do not extend into submucosa. A decision was made on December 31, 2012 to systemically adopt the technical innovation because EMS showed early success in complete removal of non-pedunculated polyps without substantial submucosal injury. This report analyzed the effectiveness of EMS in preventing postpolypectomy bleeding and facilitating complete polyp removal, especially for high-risk advanced polyps.

## Methods

### Patients and study design

This was a retrospective investigation of data incorporating electronic medical record and pathology slides at Minnesota Gastroenterology, PA (MNGI). An Internal Review Board exemption was granted for the investigation. The patients were those who had colonoscopy with snare polypectomy under Current Procedural Terminology (CPT) code 45385 by ZJC at MNGI ambulatory endoscopy centers during a 12-year period divided into pre-EMS era (2005–2012) and EMS era (2013–2016). A database search was performed for postpolypectomy bleeding cases requiring hospitalization and advanced polyps defined as polyps with villous features, high-grade dysplasia, sessile serrated adenoma with cytological dysplasia (SSACD) as well as any adenomatous polyps 10 mm or larger. Because MNGI had extensive local practice coverage and a 24-h staffed complication telephone line, it had an almost complete catch rate for complications which were peer-reviewed and fed-back to the involved endoscopists for quality improvement.

### Endoscopic mucosal stripping (EMS)

**Figure 1:**
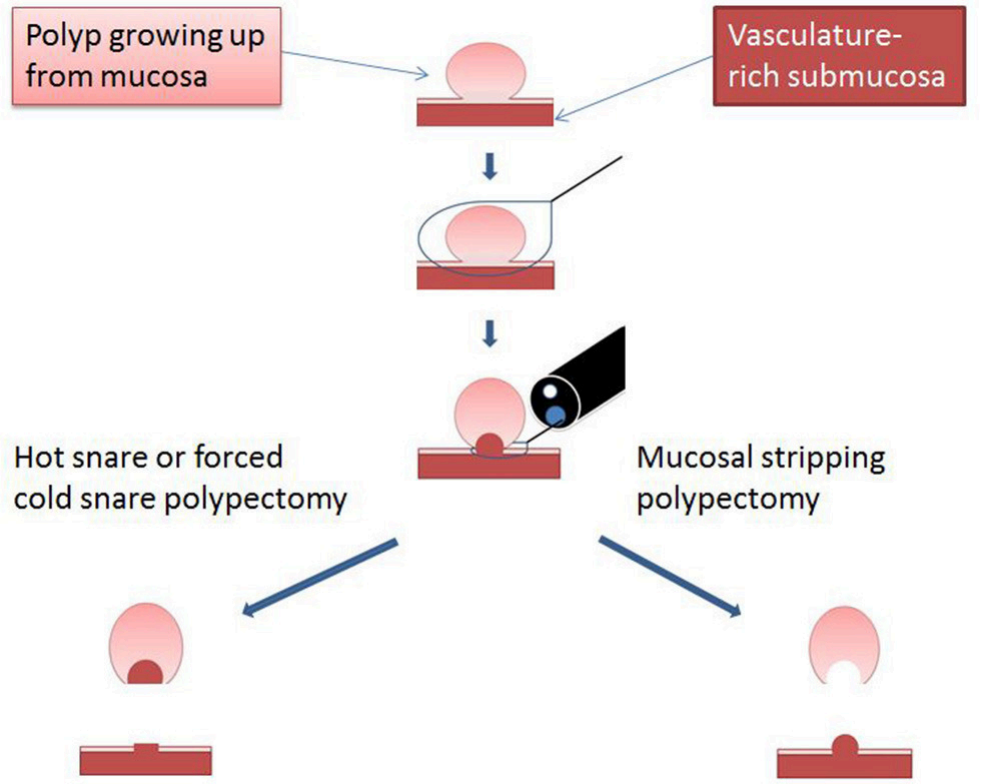
Diagram illustrating the critical difference between EMS and traditional snare polypectomy. Reprinted from Chen and Batts (38), Copyright (2017) with permission from Elsevier for use as open access content under CC-BY user license.

Endoscopic mucosal stripping (EMS) is the endoscopic removal of polyp-containing mucosa by mechanical stripping using a cold snare. It is a modification of TCSP in that EMS strips the mucosa off the ensnared submucosa at the potential space between them (Figure 1, Supplementary video) rather than guillotines the entire ensnared tissue as originally described for TCSP (36, 37). EMS is therefore expected to minimize submucosal injury and associated immediate and delayed bleeding. For the rare pedunculated polyp, we still use hot snare polypectomy because the cautery-induced tissue damage can be largely confined to the intraluminal stalk and does not extend deep into the colonic wall. Hence, we applied EMS exclusively to non-pedunculated polyps without features suggestive of malignancy. Below, we detail the specifics of EMS techniques based on the polyp size.

**Figure 2:**
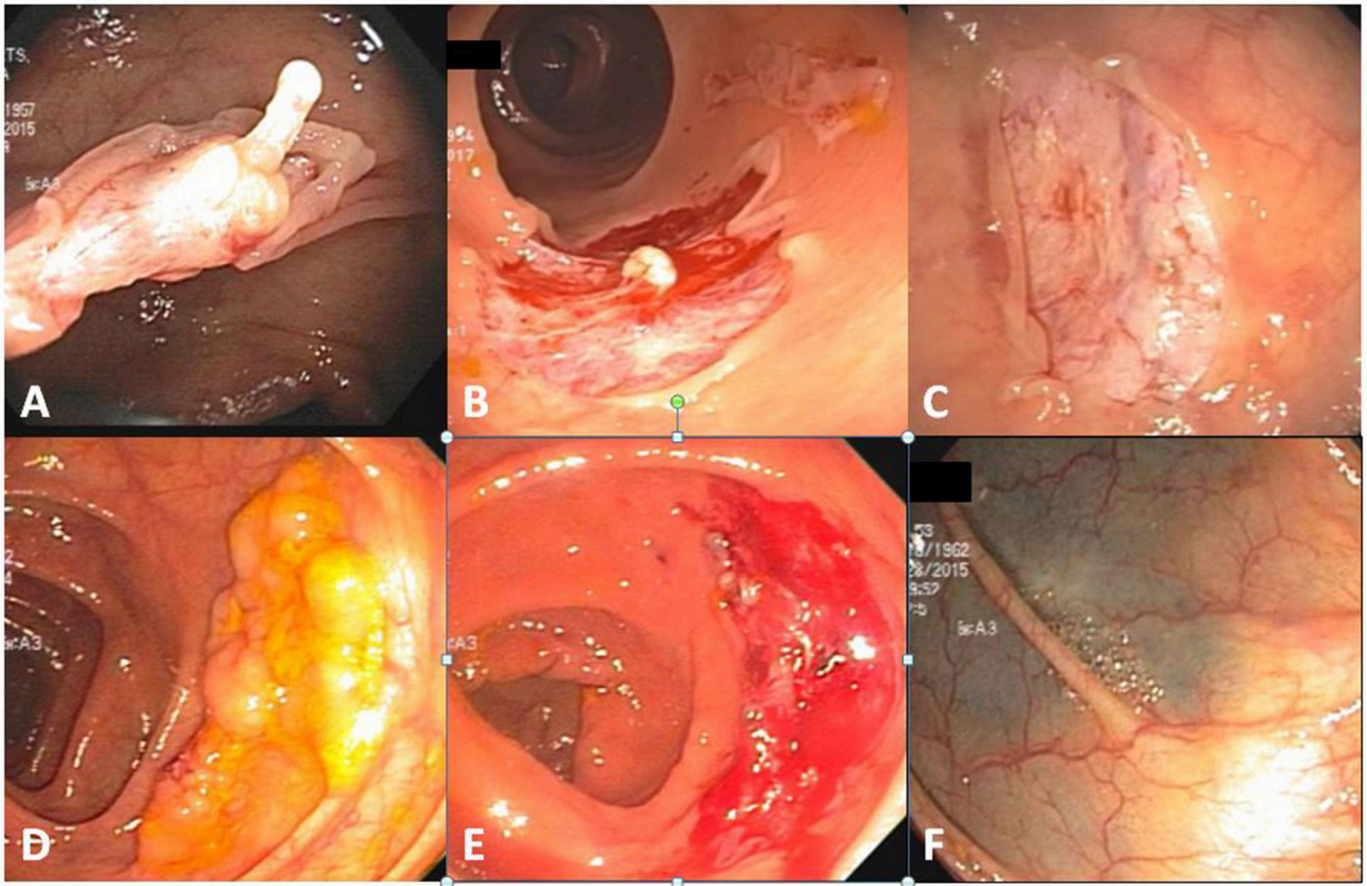
EMS polypectomies. **(A)** Submucosal pseudostalk after polypectomy of a 10-mm sessile cecal tubular adenoma. **(B)** An intact piece of polyp-containing mucosa after removal (**upper right**) and the polyp-free polypectomy site with visible pseudostalk (**lower left**). **(C)** A clean polypectomy site without deformity despite minor transient capillary oozing immediately after polypectomy. Lifting of the entrapped mucosa prevented pseudostalk development. **(D)** A 30 mm transverse colon sessile serrated adenoma. **(E)** Polypectomy site of **(D)** after piecemeal polypectomy. **(F)** Same tattooed polypectomy site as **(E)** 1 year later.

For small polyps (≤ 5 mm), TCSP generally works well. To ensure complete resection, we prefer snare over biopsy forceps except for diminutive polyps 2 mm or smaller (25) and routinely remove 2–3 mm adjacent mucosa during polypectomy. However, submucosa can still get trapped inside the snare preventing complete snare closure without brute-force guillotine or heat coagulation (hot snare). When this occurs, steady gentle pulling of the snare into the scope while keeping the snare firmly but not tightly closed usually results in the polyp-containing mucosa being stripped/pinched off, leaving behind the entrapped submucosa forming a transiently raised pseudostalk (Figure 2A). Endoscopically, the stripped-off mucosa is usually in one piece whereas the submucosa remains largely intact without blood vessel damage (Figure 2B). The mucosal tear caused by EMS generally creates a polypectomy site slightly larger but much less bloody than would be caused by TCSP. For EMS to be effective, it is critical to keep the snare firmly but not too tightly closed during stripping. Closing the snare not firmly enough will cause snare slip while closing the snare too tightly will lead to submucosal damage such as hematoma formation and stripping failure. An experienced endoscopist can easily sense the subtlety and instruct assisting endoscopy nurse to adjust the appropriate firmness of the grip when pulling the snare.

While submucosal entrapment can be avoided for smaller polyps by optimally placing the snare and gently lifting the polyp, this may become inevitable with larger polyp removal. Although the above described technique still applies, the entrapped submucosa often prevents separation of snare from polypectomy site even after the polyp-containing mucosa has already been stripped off. Under such circumstances, there is no need to further pull the snare by brute force. Instead, opening the snare slightly and sliding it over the entrapped submucosa will release the snare and any stripped off yet still seemingly attached polyp-containing mucosa from the polypectomy site. The entrapped submucosa from these larger polyps frequently forms a thicker pseudostalk but submucosal injury remains minimal.

For even larger polyps that cannot be removed in one piece, EMS piecemeal polypectomy can be performed. Because EMS' lack of submucosal injury creates a less bloody or deformed polypectomy site in comparison with traditional cold or hot snare polypectomy, any residual polyp tissue is better visualized facilitating complete polyp removal (Figure 2).

### Outcome assessment

1. Hospitalization-requiring postpolypectomy bleeding rates of all and non-pedunculated colon polyps before and after EMS adoption.
2. Completeness of EMS polyp removal determined by polypectomy site biopsy and examination during follow-up colonoscopy at intervals recommended by national society guidelines (13, 14).

### Colonoscopy examination

Colonoscopies were performed using variable stiffness colonoscopes (PCF 160, CF 160, PCF 180, and CF 180; Olympus Corp. Tokyo, Japan) for most cases and FUSE full spectrum colonoscopes (EndoChoice, Alpharetta, Georgia) for a few cases as part of a trial. Polypectomies were performed using mostly oval snares (100600 through 100602, ConMed, Utica, NY) and, in a few cases, spiral snare (SD-230U-20, Olympus Corp, Tokyo, Japan) and Exacto snare (BX00711115, US Endoscopy, Mentor, OH).

As part of MNGI quality measurement, the following annual colonoscopy statistics were reported for ZJC for 2014 and 2016: cecum reach rate 99.5–100%, screening colonoscopy adenomatous polyp detection rates 50.3–60.1% for males and 42.1–54.0% for females.

### Statistical analysis

Statistical analysis was performed using Fisher's exact test.

## Results

### EMS prevented hospitalization-requiring postpolypectomy bleeding

A total of 5,142 colonoscopies with snare polypectomy performed by ZJC at MNGI ambulatory endoscopy centers during 12 years were divided into pre-EMS era (2005–2012, *n* = 2973) and EMS era (2013–2016, *n* = 2169) (Table 1). The last hospitalization-requiring postpolypectomy bleeding occurred in September 2012. None was found during EMS era (rate 0%) whereas ten (including 2 pedunculated polyps) were found during pre-EMS era (rate 0.336%). Hospitalization occurred 1–16 days after colonoscopies. This difference was statistically significant (*P* = 0.0055) and remained so after excluding the 2 pedunculated polyp cases (*P* = 0.012).

**Table 1:** Postpolypectomy bleeding cases and total snare polypectomy (CPT code #45385) cases in 12 years.

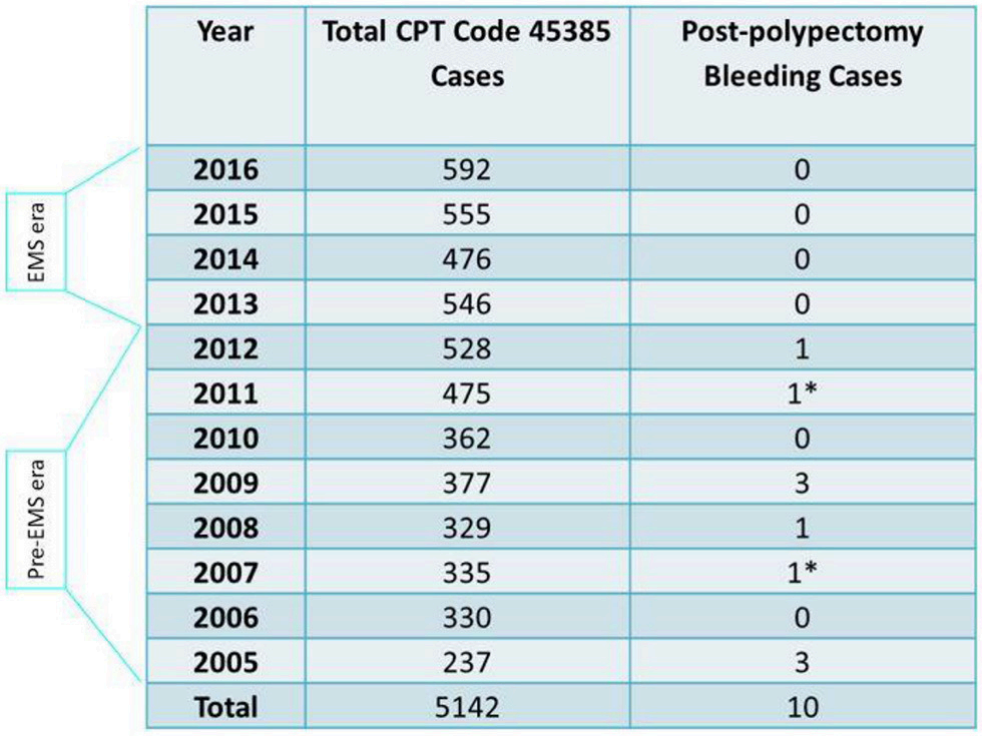

The postpolypectomy bleeding hospitalization rate of 2013–2016 (EMS era) is significantly reduced compared with that of 2005–2012 (pre-EMS era) (P < 0.0041). The significance remains (P < 0.012) after excluding the 2 bleeding pedunculated cases (marked with *).

**Figure 3:**
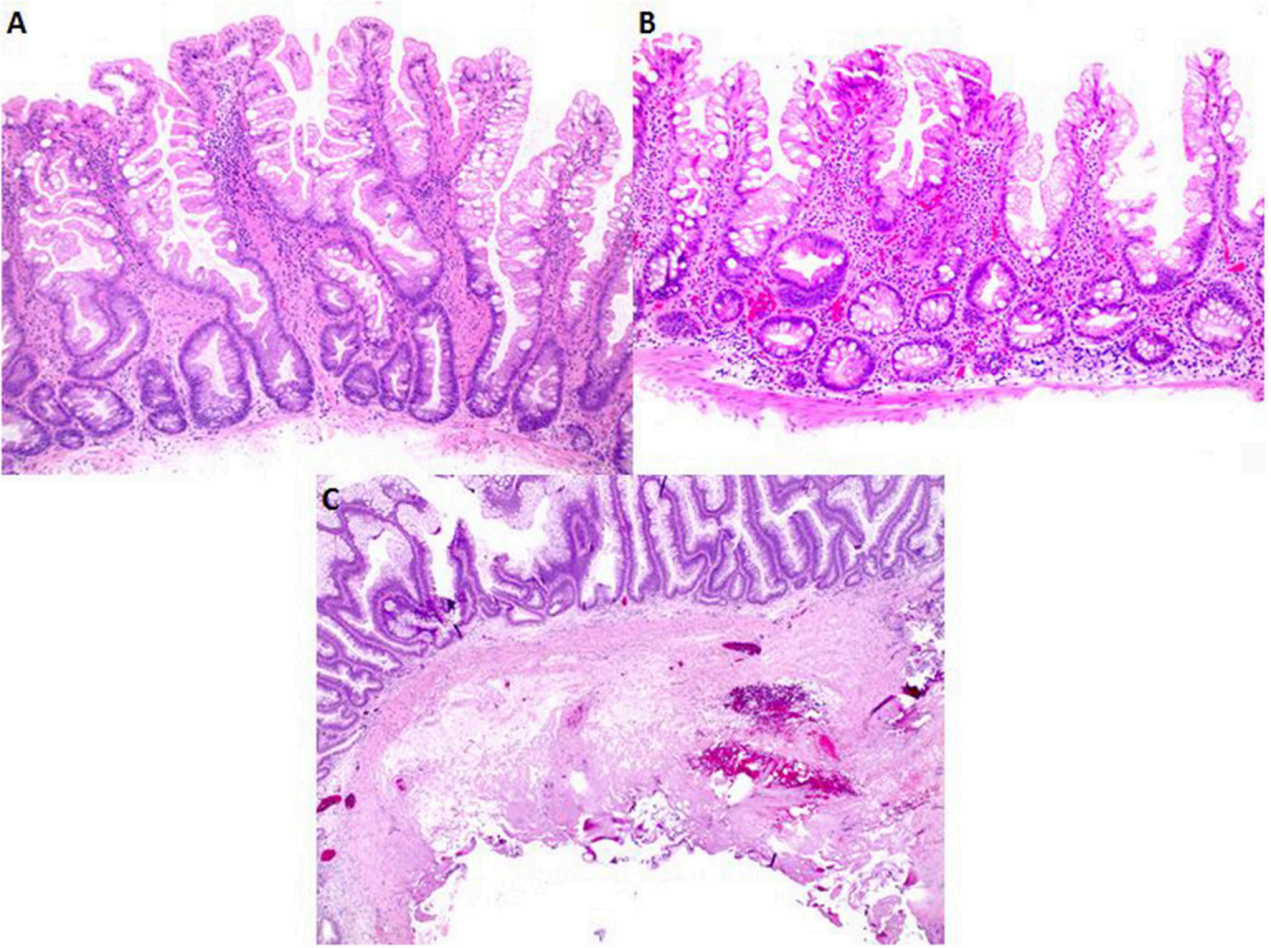
Histopathology comparison of advanced polyps removed with EMS and hot snare polypectomy. **(A,B)** Polyps removed using EMS typically show a deep aspect of muscularis mucosae with a paucity of submucosa and lack of blood vessels. **(C)** Low magnification of a polyp removed using hot snare shows typical amount of submucosa with tissue damage caused by cautery.

Of the 12 bleeding cases, a total of 18 polyps were removed. Except for two 2–3 mm tubular adenomas (TA) that were removed using a cold snare, all were removed using a hot snare. These included ten 8–40 mm TAs (including 2 pedunculated polyps of 11–15 mm and 36–40 mm, respectively), one 25 mm tubulovillous adenoma (TVA), four 6–10 mm sessile serrated adenomas (SSA) and one 5 mm lipoma. Except for one case which only had partial retrieval of the resected polyp and had no submucosa microscopically, all remaining nine cases showed evidence of cauterized submucosa often with visible blood vessels near cauterized edge as illustrated in Figure 3C. These findings indicate that submucosal damage during hot snare polypectomy contributed to postpolypectomy bleeding.

For comparison, we also microscopically examined 20 consecutive cases of advanced colon polyps removed using EMS. These polyps contained only variable amount of muscularis mucosae and scanty, if any, submucosa with no blood vessels (Table 2, Figures 3A,B). These findings indicate that EMS prevented postpolypectomy bleeding by avoiding submucosal damage.

**Table 2:** Characteristics of 20 consecutive cases of advanced colon polyps removed using EMS.

**Polyps**	**Presence of normal surrounding mucosa**	**Quartile percentage of muscularis mucosae present**	**Presence of submucosa**	**Presence of blood vessel**
TA10	+	25	–	–
TA12	+	50	–	–
TA10	+	25	–	–
SSA10	+	25	–	–
SSA25	+	25	–	–
TVA10	+	50	–	–
TVA15	+	75	–	–
SSA36-40	+	50	–	–
SSA26-30	+	75	Scanty	–
TA10	+	50	–	–
TA11-15	+	75	–	–
SSACD11-15	+	50	–	–
SSACD10	+	50	–	–
TA25	+	25	–	–
TA11-15	+	25	–	–
TA10-50^*^	+	50	–	–
TA21-35^*^	+	75	Scanty	–
TA35-40	+	50	–	–
TA16-20	+	25	–	–
SSA20	+	50	Scanty	–

*TA, tubular adenoma; SSA, sessile serrated adenoma; TVA, tubulovillous adenoma; SSACD, sessile serrated adenoma with cytological dysplasia. The number following the abbreviation denotes the size of polyps in mm*.

* *Multiple advanced polyps*.

### EMS facilitated complete polypectomy

**Figure 4:**
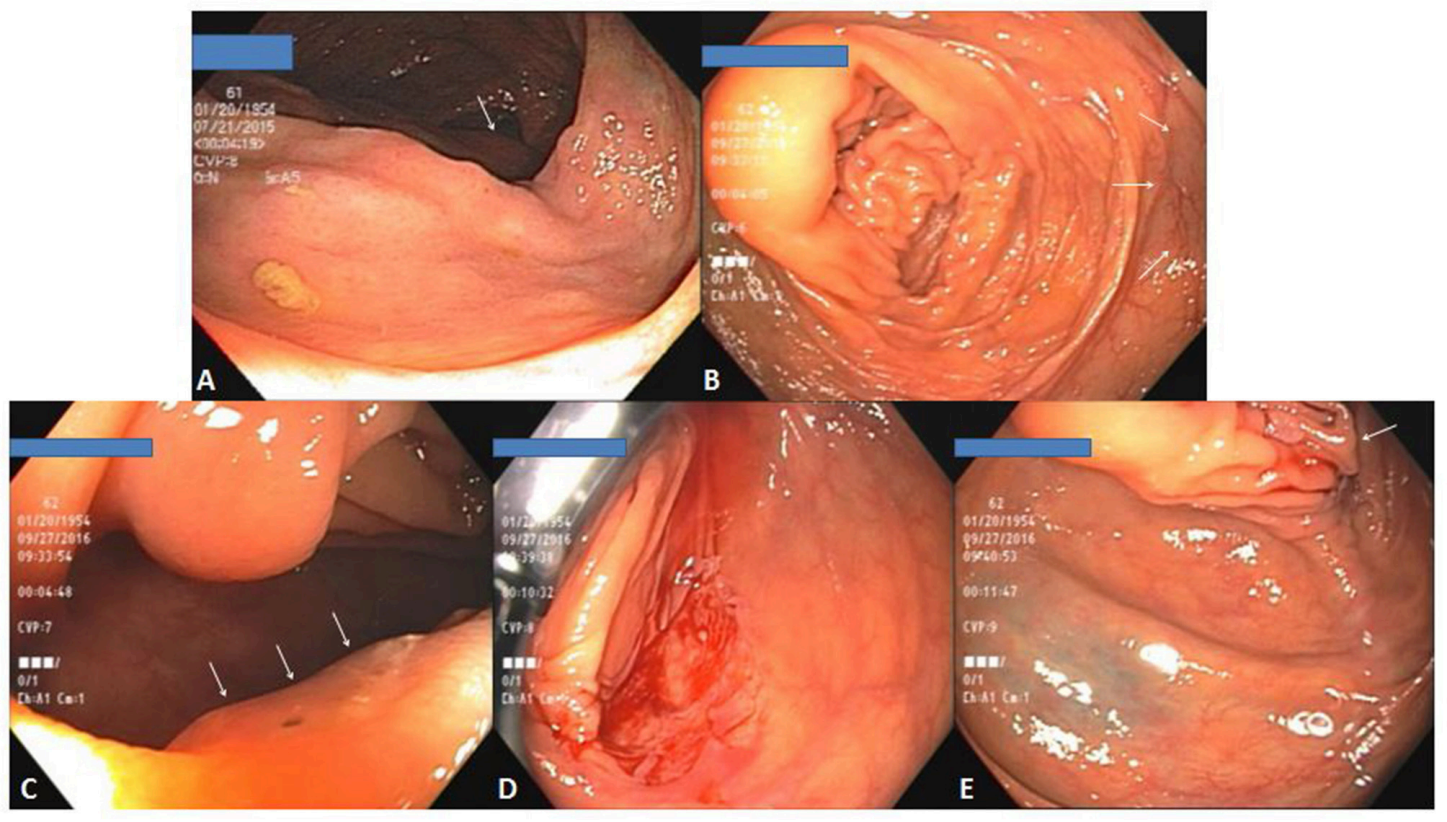
**(A)** A large 50-mm sessile serrated adenoma (Polyp#26 in Table 3) in proximal ascending colon with ICV visible in background (arrow). **(B)** Overview of the polypectomy site 14 months later with one of the tattoos visible on the right (arrows). **(C)** A 10-mm SSA hidden behind a fold (arrows) close to polypectomy site. **(D)** Polypectomy site of the polyp in **(C)** on retroflexion view. **(E)** Relative space of one of the tattoos (left lower) and the fold behind which the polyp in **(C)** hid (arrow).

Extensive biopsies were performed on the edge (x4) and pseudostalk (x1) of 10 randomly selected advanced polyp sites after endoscopically complete removal by EMS. No polyp residue was found in any samples. Ninety-seven neoplastic EMS polypectomy sites were followed from 6 months to 4 years, including 38 polypectomy sites in 23 patients after removal of advanced polyps. None of the 59 non-advanced neoplastic polyp (< 10 mm and without advanced features) sites had any residual or recurrent polyps on follow-up colonoscopy. The vast majority (33 of 38; 87%) advanced polyp sites also had no polyps on follow-up colonoscopy (Table 3). Two small polyps were found near polyp #12 site 1 year later but were of a different type than the original (SSA vs. TA). For polyps #13, #22, and #25, although follow-up colonoscopies 1–3.5 years later did find one small (2–5 mm) same type of polyp (TA) near the original polypectomy site in each case, it was difficult to determine if the polyps were newly formed or residues because they all occurred in polyposis cases with 24, 10, and 14 adenomatous polyps removed in the combined initial and follow-up colonoscopies, respectively. Different type of small polyps (SSA rather than TA) were also discovered near the original polypectomy sites of polyp #13 and #25 on follow-up colonoscopy further suggesting new polyp growth rather than incomplete polypectomy. A 10 mm SSA was found 14 months later behind a fold close to the tattooed polypectomy site of the 50-mm flat SSA (#26). This could represent incomplete polypectomy possibly because of the extra-large size of the original polyp but it could also represent an enlarging separate polyp missed in the original colonoscopy because of its very hidden location behind a fold best seen on retroflexion (Figure 4). Thus, not a single convincing case and at most 4/97 (4.1%) of all followed neoplastic polyps or 4/38 (10.5%) of the advanced polyps removed using EMS had incomplete polypectomy. These rates compared very favorably with those reported for traditional snare polypectomy techniques (18).

**Table 3:** Follow-up of 38 advanced colon polyps removed using EMS in 23 patients.

	**Colon Location**	**Shape**	**Size (mm)**	**Histology**	**Follow-up Time**	**Findings of Possible Residual Polyp**	**Note**
1	Sig	Sessile	10	TVA	2 years	None
2	HF	Flat	7	SSACD	6 months, 3.5 year	None
3	HF	Sessile	6	SSACD	1 year	None
4	HF	Sessile	50	TVA	6 month	None	Touch of hot snare^*^
5	Asc	Sessile	10	TA	3 year	None
6	Trans	Sessile	11	TA	6 month	None	Original polypectomies without stopping Plavix
7	Trans	Sessile	15	SSACD	6 month	None
8	Trans	Flat	36-40	SSA	7 month	None	Touch of APC^*^
9	Cecum	Sessile	10	TVA	2 year	None
10	Cecum	Flat	10	TA	1.5 year	None
11	Rectum	Sessile	10	TVAHD	2 year	None	UC background
12	Trans	Sessile	10	TA	1 year, 2 year	2 4-5 mm SSA at 1 year; none at 2 year	Polyposis: 24
13	Sig	Sessile	10	TA	1 year, 2 years	2 2-5 mm TA and SSA at 1 year; none at 2 year
14	HF	Sessile	8	SSACD	6 month, 4 years	None
15	HF	Sessile	10	TA	2 years	None
16	HF	Sessile	10	TA	2 years	None
17	Sig	Sessile	10	TA	1 year	None
18	Sig	Sessile	10	TA	6 months, 1.5 years	None
19	Cecum	Sessile	11–15	TVAHD	3.5 years	None
20	Asc	Sessile	10	TVAHD	3.5 years	None
21	Trans	Sessile	10	SSA	3.5 years	None
22	Sig	Sessile	10	TA	3.5 years	2–3 mm TA	Polyposis: 10
23	Cecum	Sessile	11–15	TA	4 years	None
24	Trans	Flat	10	TA	4 years	None
25	HF	Sessile	10	TA	3 years	2 3–5 mm TA and SSA	Polyposis: 14
26	Asc	Flat	50	SSA	14 months	10 mm SSA	Residual polyp behind fold close to tattooed polypectomy site
27	Cecum	Sessile	16–20	TVA	10 months	None
28	Dsc	Sessile	11–15	TA	10 months	None
29	Sig	Sessile	15	TA	10 months	None
30	Cecum	Sessile	10	TA	1 year	None
31	Asc	Sessile	15	TA	1 year	None
32	Asc	Sessile	11	TA	1 year	None
33	HF	Sessile	10	TA	1 year	None
34	Dsc	Sessile	10	TA	1 year	None
35	Sig	Sessile	10	TA	1 year	None
36	Rectum	Sessile	11–15	TVA	1 year	None
37	Cecum	Flat	26–30	SSA	7 months	None	Original polypectomies using EMS by a colleague
38	Asc	Sessile	15	SSA	7 months	None

*Sig, sigmoid; HF, hepatic flexure; Asc, ascending colon; Trans, transverse; Dsc, descending; TVA(HD), tubulovillous adenoma (with high-grade dysplasia); SSA(CD), sessile serrated adenoma (with cytological dysplasia); TA, tubular adenoma; APC, argon plasma coagulation*.

* *The tip of a hot snare and APC were used for removing small suspected polyp residue in #4 and #8, respectively*.

## Discussion

Our data analyses confirm EMS prevented postpolypectomy bleeding and facilitated complete polyp removal compared with traditional snare polypectomy techniques. EMS therefore could make colonoscopy safer and more effective as a colon cancer prevention tool.

Colonoscopy complications such as postpolypectomy bleeding and perforation occur because of injury to submucosa and deeper tissues. Such injury is not necessary for removing precancerous colon polyps and should be avoided.

**Figure 5:**
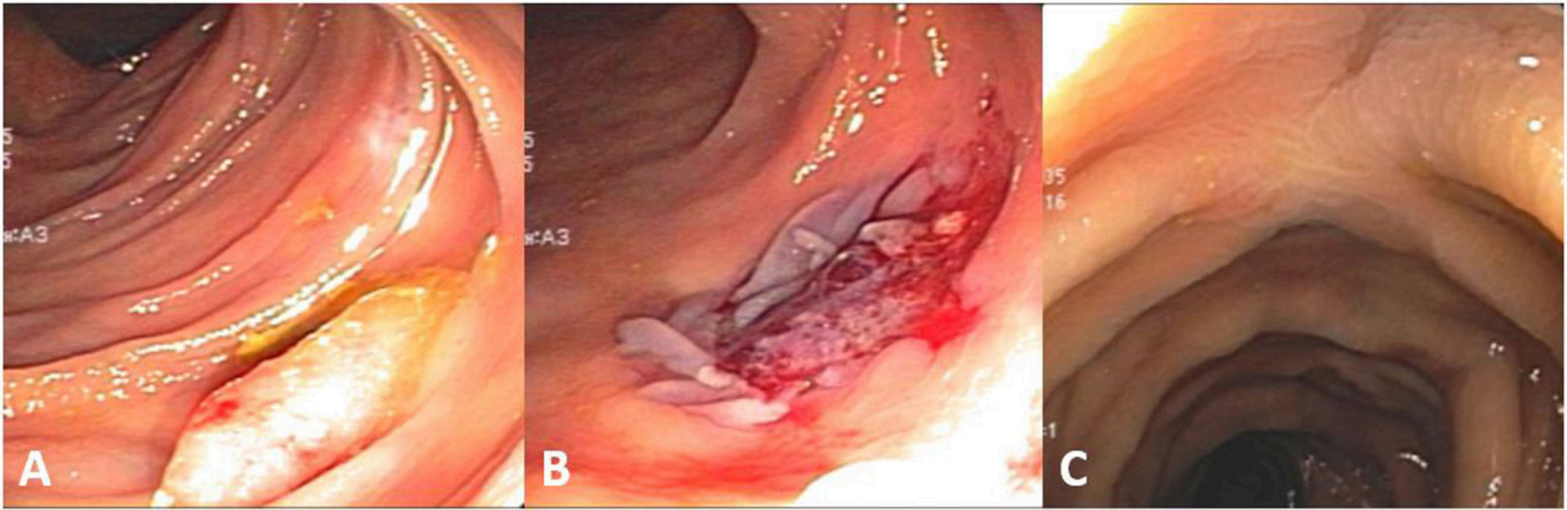
**(A)** A 15-mm transverse colon sessile serrated adenoma with cytological dysplasia (Polyp#7 in Table 3). **(B)** Polypectomy site immediately after removal of polyp in **(A)**. **(C)** Same polypectomy site as **(B)** 6 months later. This patient did not stop Clopidogrel at the time of polypectomy. With the safety profile of EMS, a decision was made to proceed with polypectomy considering that she might have a difficult repeat colonoscopy because of her advanced age (4 months shy of age 80) and a long and tortuous colon with constipation. She did eventually have a colonoscopy 6 months later for the polyp's high-risk histopathology.

Submucosa is rich in connective tissue and blood vessels. Its entrapment often occurs during snare polypectomy preventing complete snare closure without brute-force guillotine or heat coagulation. TCSP technique to guillotine the entire ensnared tissue damages entrapped submucosa although to a lesser extent compared with hot snare technique (39). The increased immediate bleeding rate with TCSP requiring hemostatic intervention such as clipping not only could impair polyp residue detection but also restricts its application to larger polyps (39–42). By avoiding cutting submucosa entirely, EMS prevents immediate and delayed bleeding and appears safe for larger polyps even without stopping anticoagulation and antiplatelet therapies (Figure 5).

A potential concern is EMS polypectomy completeness, especially in the pseudostalk. Our polypectomy site biopsy data and others' analysis of pseudostalk after cold snare polypectomy (43) showed complete polyp removal. This is further supported by the frequency with which we detected possible residual polyps at EMS polypectomy sites during follow-up colonoscopy (likely 0% and at most 4.1% all neoplastic or 10.5% advanced polyps) which is much lower than previously reported (18). Based on samplings of the edges of the endoscopically “complete” polypectomies, Pohl et al noted unexpectedly persistent neoplasms in 10.1% of all polypectomies, 17.3 % of 10–20 mm neoplastic polyps, greater rates for SSAs than for TAs (31 vs. 7.2%), and 47.6% of all large (10–20 mm) SSAs were incompletely removed (18). Zhang et al. similarly reported incomplete TCSP of polyps (6–9 mm) (8.5% on average and 13% for piecemeal resection) with immediate bleeding requiring clipping occurring at 1.8 and 2.7% per-polyp and per-patient rate, respectively (40). A probable explanation for the difference is that EMS polypectomy sites were less traumatic with minimal bleeding or deformation compared with those of traditional cold or hot snare polypectomy. This not only facilitated detection and removal of polyp residue but also allowed more generous mucosal resection without concerns for bleeding and other complications. Complete polypectomy is important in preventing interval colon cancers which account for 2.6–9.0% of total colon cancers (19).

Pedunculated polyps may have a high risk of postpolypectomy bleeding because submucosal injury is inevitable. Gain of experience may have led to reduced bleeding from these rare polyps as ZJC shifted the cutting away from the colon wall and close to the polyp on the stalk. But the reduction in postpolypectomy bleeding from pedunculated polyps before and after EMS was not statistically significant. We believe EMS rather than gain of experience led to the elimination of postpolypectomy bleeding from non-pedunculated polyps.

A hematoma could rarely develop from a torn submucosal vessel during EMS of large polyps. However, the intact submucosa keeps any hematoma from enlarging and exsanguinating. In our experience, the natural barrier provided by intact submucosa appeared superior to coagulation provided by hot snare in preventing delayed postpolypectomy bleeding.

Compared with EMR, EMS is much easier to perform and may achieve the same polypectomy result even for some large lateral spreading lesions (Table 3) without additional resources and with much reduced bleeding risk. Further studies are needed to compare EMS and EMR directly, especially for removal of large polyps.

Because EMS only removes mucosa, any early cancer with submucosal invasion is beyond its scope. Of note, we did encounter one case of a 10 mm unsuspected cancerous colon sessile polyp during the study period. Interestingly, the polypectomy site continued to ooze and required hemoclipping. The diagnosis was obtained only after polyp removal and the patient was subsequently referred to surgery without incidence. Magnified pit pattern diagnosis may have been helpful in a prediagnosis in this case and careful examination of the polyp is always encouraged before EMS to avoid cutting cancerous polyp. However, no adverse outcome occurred in this case.

EMS may be performed with spiral or Exacto snares and in combination with submucosal injection for mucosa lift, argon plasma coagulation or hot snare tip ablation. It remains to be seen whether they further improve EMS efficacy.

EMS has been successfully adopted by quite a few MNGI colleagues (Table 3, #37–38) but the generalizability of its benefits needs further confirmation studies. Nevertheless, this relatively simple yet effective innovation in polypectomy technique could represent a new frontier for safer and more efficacious colon cancer prevention by colonoscopy.

## Ethics statement

This retrospective study was granted Exemption from IRB Review Determination on May 12, 2016 (IRB ID: 5502) by Sterling IRB (www.sterlingirb.com) and was carried out in accordance with the approved protocol of Clinical and Histopathological Review of Mucosal Stripping as a Colonoscopy Polypectomy Technical Innovation and the recommendations of Minnesota Gastroenterology Quality Committee. All subjects gave written informed consent to the colonoscopy procedures and treatment in accordance with the Declaration of Helsinki.

## Author contributions

ZC initiated the investigation concept and design, acquisition, analysis and interpretation of data, drafting and revising of the manuscript. KB participated in the acquisition, analysis and interpretation of data as well as critical revision of the manuscript.

### Conflict of interest statement

The authors declare that the research was conducted in the absence of any commercial or financial relationships that could be construed as a potential conflict of interest.

## Abbreviations

EMS: endoscopic mucosal stripping
EMR: endoscopic mucosal resection
ESD: endoscopic submucosal dissection
TCSP: traditional cold snare polypectomy
TA: tubular adenoma
TVA(HD): tubulovillous adenoma (with high-grade dysplasia)
SSA(CD): sessile serrated adenoma (with cytological dysplasia)
UC: ulcerative colitis.
